# Driving Medical Innovation Through Interdisciplinarity: Unique Opportunities and Challenges

**DOI:** 10.3389/fmed.2019.00035

**Published:** 2019-02-26

**Authors:** Faekah Gohar, Patrick Maschmeyer, Bechara Mfarrej, Mathieu Lemaire, Lucy R. Wedderburn, Maria Grazia Roncarolo, Annet van Royen-Kerkhof

**Affiliations:** ^1^Department of Paediatrics, Clemenshospital, Münster, Germany; ^2^Therapeutic Gene Regulation, Deutsches Rheuma-Forschungszentrum (DRFZ), Institute of the Leibniz Association, Berlin, Germany; ^3^Center for Cell Therapy, Institut Paoli-Calmettes, Marseille, France; ^4^Division of Nephrology, Department of Paediatrics, The Hospital for Sick Children, University of Toronto, Toronto, ON, Canada; ^5^UK National Institute for Health Research Great Ormond Street Biomedical Research Centre, London, United Kingdom; ^6^Arthritis Research UK Centre for Adolescent Rheumatology at University College London (UCL), University College London Hospitals (UCLH) and GOSH London, London, United Kingdom; ^7^Institute for Stem Cell Biology and Regenerative Medicine, Stanford University, Palo Alto, CA, United States; ^8^Department of Pediatric Immunology and Rheumatology, Wilhelmina Children's Hospital Utrecht, Utrecht, Netherlands

**Keywords:** interdiscipinarity, societal impact, translational medical research, innovation, collaboration

## Introduction

Many health problems facing society are multifactorial and often require social and political input as well as interventions from medical and technological experts. For example, the treatment of chronic pain requires expertise from multiple disciplines: imaging technology, cellular electrophysiology, neurochemistry, genetics, social, psychological, and cultural studies (1). While these activities are coordinated by the treating physician, they usually remain parallel and are never fully integrated to create an innovative therapy for the patient. From a research standpoint, we argue that for these new solutions to emerge, there needs to be a concerted effort to move from multidisciplinarity to interdisciplinarity.

Multidisciplinary research is defined as work involving researchers from different fields who “remain conceptually and methodologically anchored in their respective fields” (2). In contrast, interdisciplinary research is defined as “a mode of research by teams or individuals that integrates information, data, techniques, tools, perspectives, concepts, and/or theories from two or more disciplines or bodies of specialized knowledge, to advance fundamental understanding or to solve problems whose solutions are beyond the scope of a single discipline or area of research practice” (3). It may lead to the creation of a new scientific field, such as environmental humanities (4–6).

The major difference between the two types of research is that while interdisciplinarity involves deep and robust integration of distinct disciplines, multidisciplinarity implicates juxtaposition of a variety of expertises (5). By these definitions, both research types are clearly valuable, but interdisciplinary research should drive more impactful results for complicated problems. These advances come at a cost for researchers because interdisciplinarity has its own set of unique challenges, ranging from communication issues to allocation of credits among a team. In this article, we discuss these hurdles and potential solutions to raise awareness amongst researchers keen to lead a successful interdisciplinary project.

## Characteristics of an Interdisciplinary Team

Collaborative teams consist of individuals from different fields working toward a common goal that transcends the borders of a single discipline. Exactly who will comprise the members of an interdisciplinary and multidisciplinary team must be individually determined for each project according to the specific needs. It is almost a certainty in research projects that individuals will face hurdles that can only be solved with group support, leading to a widespread feeling among members of being out of one's comfort zone (7). Communication can be challenging when a team involves members from a variety of disciplines. A classic strategy employed to dominate the discourse and decision-making process is to use highly technical language specific to one's field of expertise. Bammer proposed the creation of a new role for integration and implementation scientists (8). Such experts would contribute to teams tackling complex problems by assessing the problems and their interconnections, and by identifying strategies for approaching them. These implementation scientists could define the level of involvement of the different stakeholders and strategize how to incorporate the various disciplines and stakeholder objectives. Furthermore, they can identify knowledge gaps and predict evolving problems, whilst providing support throughout the process. Two major hurdles can be identified: first in identifying a universal requirement for experts in this role, and secondly establishing a clear identity for scientists in this role with a clear consensus on methods and processes to be used for example in training for such a role (9).

In the same direction, a new field of research is developing, which was first termed “the science of team science” or SciTS in 2006. This field focuses on systematic efforts to overcome barriers in collaborative work, and how to achieve the targeted research outcomes. Other goals of SciTS are to support scientists in creating and working effectively within a team. However, above a certain team size (different for each research setting and question) output decreases and bureaucracy increases, with potential conflicts arising within teams. Therefore, in a world of limited resources, important questions for researchers also include the question of resource allocation i.e., when to decide if external collaborators or cross-disciplinary support is required and how to fund this adequately (10).

Efficient coordination of project tasks is vital for progress to occur. In large teams, a power struggle for the “lead” role may emerge when several individuals have equal seniority or leadership experience. The team leader must match responsibilities to expertise and time commitment, to plan a schedule that is realistic yet ambitious, and to provide ample opportunities for team members to share updates and knowledge. The team leader also often plays a key role in designing the research plan and in identifying potential team members with complementary knowledge and skills. “The science of team science” is a new field of research that aims to provide evidence to support scientists responsible for these tasks and helps them to overcome barriers (11).

A survey of researchers revealed that successful interdisciplinary work often includes mutual respect, comfort, or already established positive relationships (12). These concepts gave rise to a new ethical framework known as relational ethics, stemming from the fact that all ethics are grounded in relations, interdependency, engagement and the importance of community (13). This framework suggests that a climate of safety, trust, respect and equality is necessary to effectively challenge the *status quo* (14, 15).

Successful solutions to complex problems can be achieved when a team is comprised of individuals with complementary expertises, interests, ideas, and/or professional goals. An example is the creation of arterio-venous fistulas for hemodialysis access using an innovative endovascular catheter-based system: this system was conceived and implemented by a team of interventional radiologists, vascular surgeons, biomedical, and industrial engineers (16). Another example is the invention of a blood-resistant biodegradable surgical glue by a team of pediatric cardiologists, cardiac surgeons, biomedical, biological, and chemical engineers (12). In both cases, long-identified unmet medical needs became solvable because of well-directed interdisciplinary efforts over many years.

## Advantages and Hurdles of Working in Interdisciplinary Projects

The interpretation of the concept interdisciplinarity varies among individuals. It is reported that researchers face challenges in justifying the benefit of interdisciplinary interactions against their perception of increased time and resource requirements. In a study by Roy et al. both natural and social scientists identified departmental or institutional difficulties, communication difficulties and differing disciplinary approaches as significant challenges (17).

In another descriptive study, 19 researchers indicated that they conducted interdisciplinary research specifically because of their individual lack of knowledge in some sectors (7). Other benefits were the generation of new knowledge, exposure to new methods or theories, and the opportunity to make a bigger impact. However, the respondents also indicated caveats to performing interdisciplinary work, such as the need to allocate more time compared to their usual line of research as well as limited credits for academic promotion. Other issues highlighted included the significantly greater effort needed to understand interpersonal dynamics, to clarify leadership roles, and to determine the contributions of each team member. Finally, some researchers noted that some individuals may be marginalized as a result of power imbalances (18).

Funding agencies have traditionally rewarded independent scientists proposing research in their field of expertise rather than teams of researchers offering to conduct interdisciplinary projects. Over time, complex problems such as climate change led to increased funding for inter- or multidisciplinary research teams. Some researchers have argued that efforts to make research funding contingent on inclusion of interdisciplinarity leads to inefficiency (7). How successful such interdisciplinary focused funding approaches are remains unclear: the US National Institutes of Health (NIH) reports slightly better outcomes for funding fostering interdisciplinary funded programmes vs. conventional, projects of independent research, whereas the opposite is true for the European Research Council (ERC) (18). Funding for collaborative projects are increasingly available and are internationally well supported. For example, the European Framework Program for Research and Innovation, which includes the “Horizon 2020” (H2020) program, is the world's largest interdisciplinary funding program (19). In the USA, the National Science Foundation (NSF) (20) and the Clinical and Translational Science Awards (CTSA) Program supports national networks of medical research institutions that collaborate to improve the efficiency of translational research, promoting the integration of underserved populations, and train future translational researchers (21).

**Figure 1:**
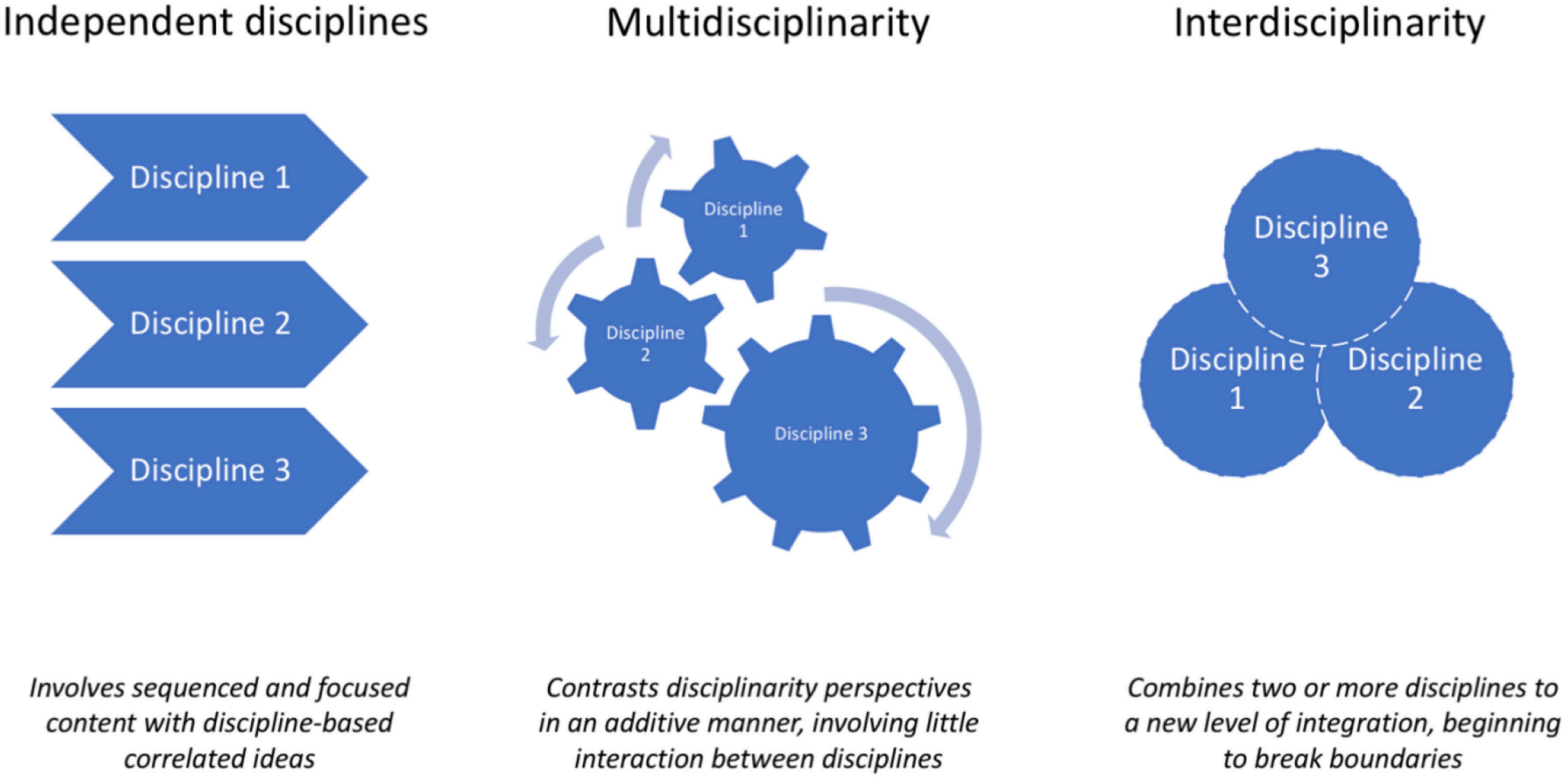
Definitions and illustrations of independent, multidisciplinary and interdisciplinary working.

In summary, many researchers hold negative perceptions about interdisciplinary research. However, these perceptions could be overcome by adopting strategies such as advanced planning of the study, including whether a project is to be multi- or interdisciplinary (see Figure 1), and by including a balanced team with the abilities required for the project (see Table 1).

**Table 1:** Recommendations to stimulate sustainable interdisciplinary research environments.

**Pre-project**
Include a trainee or have a future team member seek additional training in a program with a focus on interdisciplinary research.
Determine the extent of collaboration wished (inter- vs. multi-disciplinary).
Plan the team composition, the balance of abilities and role delegation. Consider including a scientist in an integration and implementation role.
**During the project**
Allocate the supervisor role to someone with experience of interdisciplinary project supervision, not necessarily the most senior.
Plan early for potential project hurdles, such as funding issues, allocation of funds, credits etc.
Plan the allocation of credits, such as the authorship order, early.
Focus on the training of inexperienced project members.
Consider the implementation of a team-based mentoring program and integrate team-based evaluation.
**Post-project**
Ask for anonymous feedback from all team members on what worked well and what could be done better to provide helpful hints to improve the team performance.
Consider success of the project to be not only based upon achievement of publication in high-impact journals, but rather achievement of societal goals and wider translational objectives.
All team members actively engage in knowledge translation to promote the project in their own field, including considering the use of “newer” resources or publication modes such as interactive Journals or Social Media.

## Interdisciplinary Research in Early Career Stages and for Career Progression

The World Health Organization (WHO) has recently concluded that effective interdisciplinary education facilitates later collaborative practice (22). Introducing the interdisciplinarity concept early in a scientist's career promotes the later unconscious incorporation of it into their future research (23). As a result, this early practical exposure ensures the new generation of researchers is better equipped to manage the challenges of interdisciplinarity. The integration of interdisciplinarity into higher education could be driven by educational institutions, the UK Research Excellence Framework being a good model (24).

A more structured approach is the formation of multidisciplinary translational teams (MTT) as a training and mentoring approach focusing on translational innovation by research capacity building, interprofessional integration, and team-based mentoring approaches. This methodology enhances the development of translational research competencies and productivity in terms of collaborative publications (25, 26). Another innovative structured approach is industry-based studentships, as recommended by the Canadian Academy of Health Sciences (CAHS) after an in-depth assessment of interdisciplinary health research (27). An argument against this model of training is that it increases pressure and constraints placed on trainees by adding an additional layer of training and evaluation to their portfolio.

For challenging topics with dedicated grants and that require interdisciplinary approaches, evaluation of teams supersedes the evaluation of individuals. Yet, the coordinator carries most of the evaluation pressure, since their track record needs to show they have coordinated interdisciplinary teams and trained next-generation scientists to implement interdisciplinary research. It is true that progression from early stage to established scientists requires continuous evaluation with the “expertise” binoculars, yet one needs to start somewhere. The pressure is on early-stage researchers to acquire “expertise” in order to progress, yet, be open to learning and implementing interdisciplinary methods in preparation for the tackling of complex problems.

Throughout their careers, scientists are traditionally evaluated based on the quality of their output. Articles only “count” in the academic tally if the scientist is first or last author. Middle authorships are reflexively disqualified irrespective of the nature of the contribution or the importance of the discovery. Scientists interested to work as part of interdisciplinary teams may be discouraged to do so when realizing that they will be at a significant disadvantage compared to others who prefer “flying solo.” McLeish and Strang identify “Individual Career Progression” as one of the crucial levels at which there is an immediate need for an effective evaluation method for interdisciplinarity (28).

Furthermore, from their experience as evaluators, the authors report enormous pressures on researchers to establish a distinct identity, fueling the claim that career progression is hampered by interdisciplinary research and potentiated by single-discipline work. Nevertheless, some successful interdisciplinary translational researchers counter-argue that their aim is impact, a goal favored by several institutions. “*Resisting the concept of focusing in research meant to surround myself with collaborators of different skills to fill the gaps in knowledge and exploring constantly new areas. One's focus gets defined by products* (29) *and technologies they put on the market that have large impact on patients' lives*”—personal communication, Dr. Jeffrey Karp from Brigham and Women's Hospital, Boston (MA) (30).

## What is the Best Approach for Training Future Scientists?

While it is critical to continue training scientists who are highly knowledgeable in one specific field, it is important to expose them early on to the notions of multi- and inter-disciplinarity. Ideally, this exposure would be an integral part of their didactic and practical training. It is also critical to strive to train individuals with broader interests by allowing them to straddle a few fields during their training, with the understanding that their training is likely to be substantially longer than usual (and thus will require unusually long periods of support from funding bodies). The clinician-scientist training model is an example of this approach since it generates a workforce that is conversant in the language of both clinical and basic science. This will facilitate the dialogue between the disciplines and render a deeper mutual understanding. There are now a large number of training programs for non-physicians that aim to specifically train researchers focused on interdisciplinarity in a given discipline such as cancer or cardiovascular diseases, although no specific standards for training exist to which these programs can be evaluated by.

## Independent vs. Team vs. Interdisciplinary Science

It is important to emphasize that our goal is not to dismiss independent or team science. These two approaches, which rely on work within a more narrow scientific perspective, are distinguished by the number of independent teams involved. There are many important research questions that are best addressed using either of these traditional approaches. For example, assessing the impact of a genetic deficiency on human physiology using genetically engineered cellular or animal models. Reductionism is often a critical heuristic device to solve these scientific problems. In contrast, interdisciplinary science is most useful to answer research questions nested in complex structures. By definition, they cannot be answered by relying only on reductionistic methods but rather require integrated, multi-pronged approaches. For example, multifactorial conditions that are caused by the confluence of multiple genetic and environmental factors have been notoriously difficult to study. This has long been a frustrating situation since many diseases under this banner are prime public health problems (e.g., diabetes, atheroembolism, hypertension, or dementia). While there is no guarantee of success, the fresh look provided by interdisciplinary science is likely to yield insights and breakthroughs that may not be otherwise possible.

## Conclusion

Whilst remembering the overarching goal of interdisciplinarity research is impact, research teams should be carefully constructed, led, and organized to allow for the fulfillment of individual objectives required for personal development, as well as for overall project success and achievement of the project aims. Effective collaborative practices are enabled by effective interdisciplinary education and can be promoted by the active provision of funding streams, in order to drive creative interdisciplinarity in academia.

## Author Contributions

FG, PM, BM, and ML made equal contributions in writing the paper. LW, MR, and AvR-K also wrote the manuscript and supervised the project.

### Conflict of Interest Statement

The authors declare that the research was conducted in the absence of any commercial or financial relationships that could be construed as a potential conflict of interest. The handling editor declared a shared affiliation, though no other collaboration, with one of the authors ML.
